# Metagenomic biodiversity assessment within an offshore wind farm

**DOI:** 10.1038/s41598-025-01541-x

**Published:** 2025-05-14

**Authors:** Phanu Theodore Serivichyaswat, Thijs Scholte, Tim Wilms, Liv Stranddorf, Tom van der Valk

**Affiliations:** 1 Centre for Palaeogenetics, Svante Arrhenius väg 20C, 10691 Stockholm, Sweden; 2 Department of Bioinformatics and Genetics, Swedish Museum of Natural History, Stockholm, Frescativägen 40, 11418 Sweden; 3 Environment and Sustainability Unit, Vattenfall Vindkraft, 6000 Kolding, Denmark; 4 Section for Quantitative Sustainability Assessment, Department of Environmental and Resource Engineering, Technical University of Denmark, 2800 Kgs. Lyngby, Denmark; 5 Science for Life Laboratory, Tomtebodavägen 23, Solna, 17165 Sweden

**Keywords:** Conservation biology, Metagenomics

## Abstract

Environmental DNA (eDNA) analysis can be a powerful tool for monitoring biodiversity and assessing human impacts on ecosystems. In this study, we employed a genome-wide metagenomic eDNA approach to assess the marine biodiversity within and around the Horns Rev 1 offshore wind farm in the Danish North Sea. Seawater samples were collected from both within the windfarm and surrounding control sites, sequenced, and analyzed using a combination of DNA k-mer matching and alignment-based classification methods. We identified a wide range of species across the tree of life—highlighting the species richness of this marine ecosystem. Our results revealed a high degree of species diversity congruence between the wind farm and control sites. While this could suggest minimal ecological disruption of the wind farm, we cannot rule out that the influence of ocean currents and water mixing the DNA from different regions dominate the species detection. We detected bioindicator species, such as *Thalassiosira, Phaeocystis* and *Skeletonema*, which can provide insights into water quality. Our metagenomic approach also enabled us to obtain population genomics insights for species, such as the European anchovy (*Engraulis encrasicolus*) and the diatom *Rhizosolenia setigera*, and genetically confirmed the origin of the invasive Sea walnut (*Mnemiopsis leidyi*) in the North Sea. This study highlights the potential of genome-wide eDNA metagenomics as a framework for assessing marine biodiversity and detecting population-level genetic signals, contributing to informed and scalable ecosystem monitoring strategies.

## Introduction

### Ecological impacts of offshore wind farms

Wind energy, generated from wind turbines, is a renewable energy source with the potential to reduce the overall greenhouse gas emission from energy production^1^. Advances in technology, decreasing production cost, and governmental subsidies have driven the global expansion of wind energy over the past decade^2^. Although generally considered as a clean energy source, offshore wind farm installations interact with marine and coastal environments^3,4^. Previous studies have reported both positive and negative biodiversity impacts^5–7^. Positive effects primarily stem from the creation of artificial reefs, which attract marine life and restrict fishing activities, benefiting local biodiversity^8–12^. Conversely, negative effects may include marine habitat alteration^3^, barrier effects on movement and disruption in migration patterns^9,13^, underwater noise and electromagnetic fields^14^ as well as increased collision risks for seabirds and bats^15^.

Despite these potential drawbacks, offshore wind farms are often considered to have a net positive impact on ecosystem structure and function, though ecological risks vary across biogeographic regions^5–7^. As the global demand for renewable energy continues to rise, the expansion of offshore wind farms seems inevitable. However, to mitigate potential ecological consequences, each project must be evaluated based on the specific environmental characteristics and vulnerabilities of the affected region.

### Environmental DNA metagenomics for ecological assessments

Assessing the ecological impacts of offshore wind farms requires biodiversity monitoring. One increasingly effective method is environmental DNA (eDNA) analysis, which enables non-invasive detection of organisms based on genetic material shed into the environment through processes such as skin cell sloughing, waste excretion, decomposition, and reproductive cell release^16,17^. eDNA analysis has emerged as a powerful tool for biodiversity assessment, facilitating species detection and identification across diverse ecosystems^17^. In marine environments, eDNA has been successfully applied to study a wide range of aquatic organisms, including fish, amphibians, reptiles, aquatic mammals, insects, plants, and microorganisms^18–21^.

Traditional eDNA analysis relies on DNA metabarcoding, which amplifies and sequences standardized species-specific genomic regions^22^. While a powerful method for detecting species presence, metabarcoding relies on predefined marker genes, which can result in incomplete taxonomic coverage, and an inability to resolve genetic variation within species and populations^23,24^. As a result, DNA metabarcoding alone is often inadequate for comprehensive assessments of population structure, genetic diversity, and ecological interactions.

Such limitations can potentially be overcome through metagenomic sequencing, in which the DNA present in a sample is randomly sequenced, thereby capturing a more comprehensive representation of the genetic diversity in a sample^25,26^. Metagenomic approaches yield genome-wide sequence data, providing a broad taxonomic resolution and enabling population genetic insights^27^. This, in turn, allows for the identification of cryptic species, the assessment of genetic diversity within populations, and the inference of ecological interactions, ultimately providing a more holistic understanding of community structures^28,29^.

Here, we employed eDNA metagenomics to analyze seawater samples from the Horns Rev 1 offshore wind farm, one of the world’s oldest operational wind farms, located off the western coast of Jutland, Denmark. The wind farm, operational since 2002^30^, offers a unique setting for evaluating biodiversity patterns in a long-established offshore wind farm. To distinguish potential wind farm-associated effects from broader environmental variation, we compared these samples to seawater samples collected from control sites outside the wind farm. This comparative approach provided insights into the marine ecosystem around the offshore wind farm and demonstrated the utility of genome-wide metagenomic analysis as a tool for population diversity assessments.

## Results and discussion

### Biodiversity assessment of wind farm

**Fig. 1:**
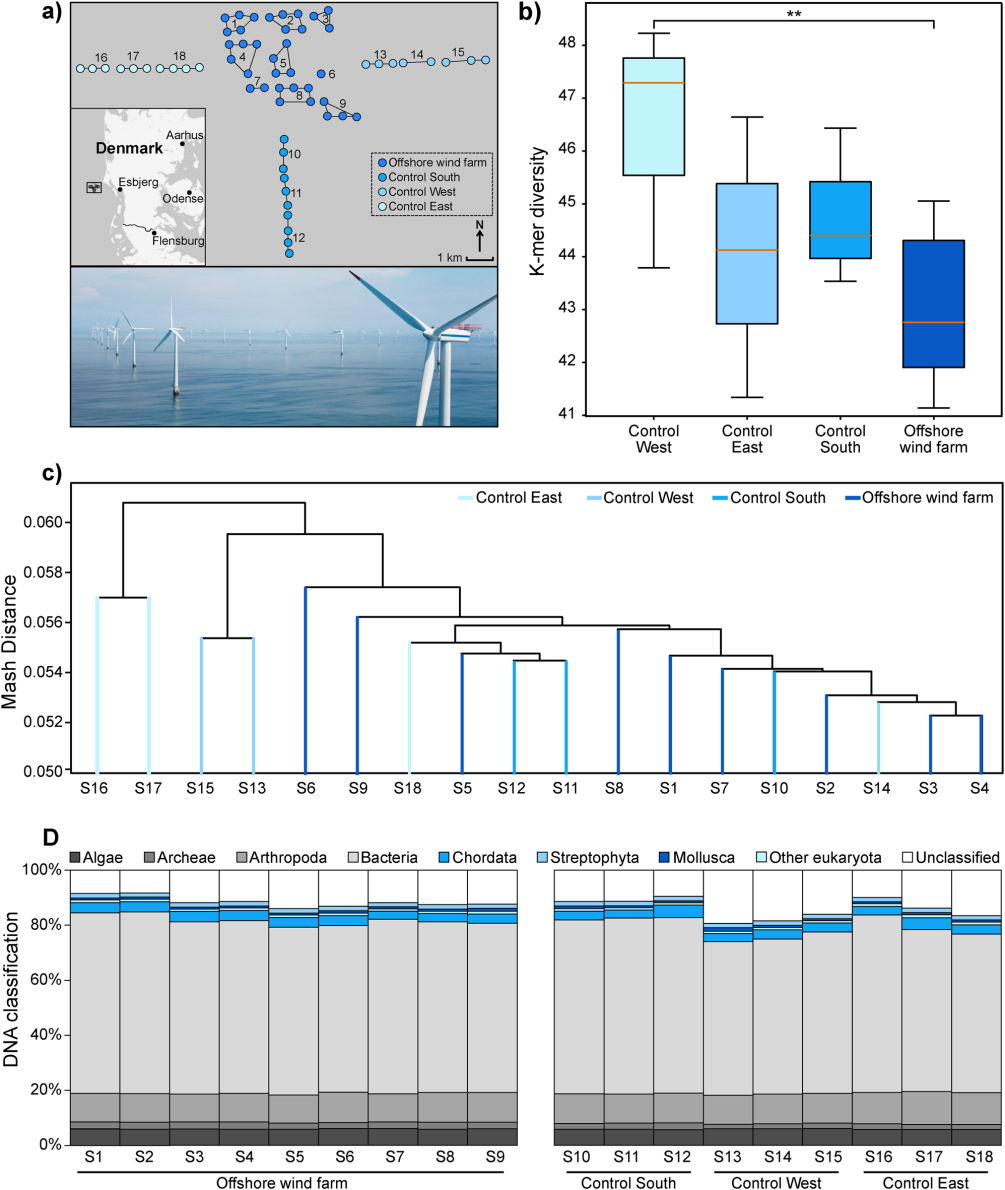
Sampling scheme and genetic diversity. (**a**) Map showing the geographical location of the total 62 seawater samples collected along the wind turbine inside the wind farm and from control sites located to the south, west, and east of the farm. Connected dots and numbers indicate the sample pooling for the DNA extraction. Scale bar = 1 km. Photo from www.powerplants.vattenfall.com/horns-rev/. (**b**) K-mer diversity between control and wind farm samples (values represent mean ± SD. *p < 0.05; **p < 0.01; ***p < 0.001; Student’s t-test). (**c**) Dendrogram showing clustering of eDNA samples. Mash distances were calculated between all pairs of samples based on all DNA reads in each sample and visualized in the dendrogram. No clustering of control and wind farm samples was observed. (**d**) Proportion of sequence reads classified to each indicated database. Between 8.3 and 19.4% of the reads remain unclassified (white).

To assess the marine biodiversity, we collected seawater from within and outside of the offshore wind farm Horns Rev 1. The collected samples were subsequently pooled for sequencing and downstream analyses according to their close approximate geographical location (Fig. 1a). We ensured comparability across pools with different sample sizes by normalizing all datasets to the same number of sequencing reads prior to the downstream analysis. Furthermore, k-mer diversity metrics showed consistent levels of genetic diversity across the sample pools, regardless of the number of samples included (Fig. S1), indicating that sample number per pool did not significantly influence overall biodiversity measurements.

In our initial analysis we quantified the number of unique 21-base pair sequences within each sample pool, based on the premise that higher species richness and species genetic variation lead to more unique DNA sequences, thus serving as a proxy for overall biodiversity. The k-mer diversity analysis revealed modest variation between sample groups, with the samples collected west of the windfarm showing significantly higher diversity (+ 10.5%) compared to samples collected from within the wind farm (Fig. 1b and Fig. S2). The samples collected in the east and south regions exhibited diversity levels similar to those of the wind farm samples, suggesting limited differences in sequence variation between sites outside and inside the wind farm. A dendrogram analysis based on genetic distance between the samples showed no distinct clustering of control and wind farm samples (Fig. 1c). Samples from all groups were interspersed, indicating no significant genetic differences between the marine communities at the different sites.

To further explore the biodiversity, we identified the species present in each sample pool by constructing comprehensive genomic databases that included reference genomes for algae, archaea, arthropoda, bacteria, chordata, mollusca, streptophyta, and other eukaryota including fish and mammals (Supplementary Tables S1-S10). Using a combined k-mer matching and alignment approach, we classified each sequence read to its most probable species of origin. The majority of sequences were assigned to bacteria (55.8–66.0%), followed by arthropods (10.1–12.0%) algae (5.7–6.1%), chordata (2.9–4.5%), archaea (1.7–2.6%), streptophyta (1.5–1.7%), mollusca (0.7–1.3%) and all other eukaryotes (0.9–1.1%) (Fig. 1d). Notably, 8.3–19.4% of the sequence data did not match any genomes in our database and was categorized as unclassified, suggesting that a subset of biodiversity in the samples comprised an unknown component of the marine life that was not captured in our analysis.

**Fig. 2:**
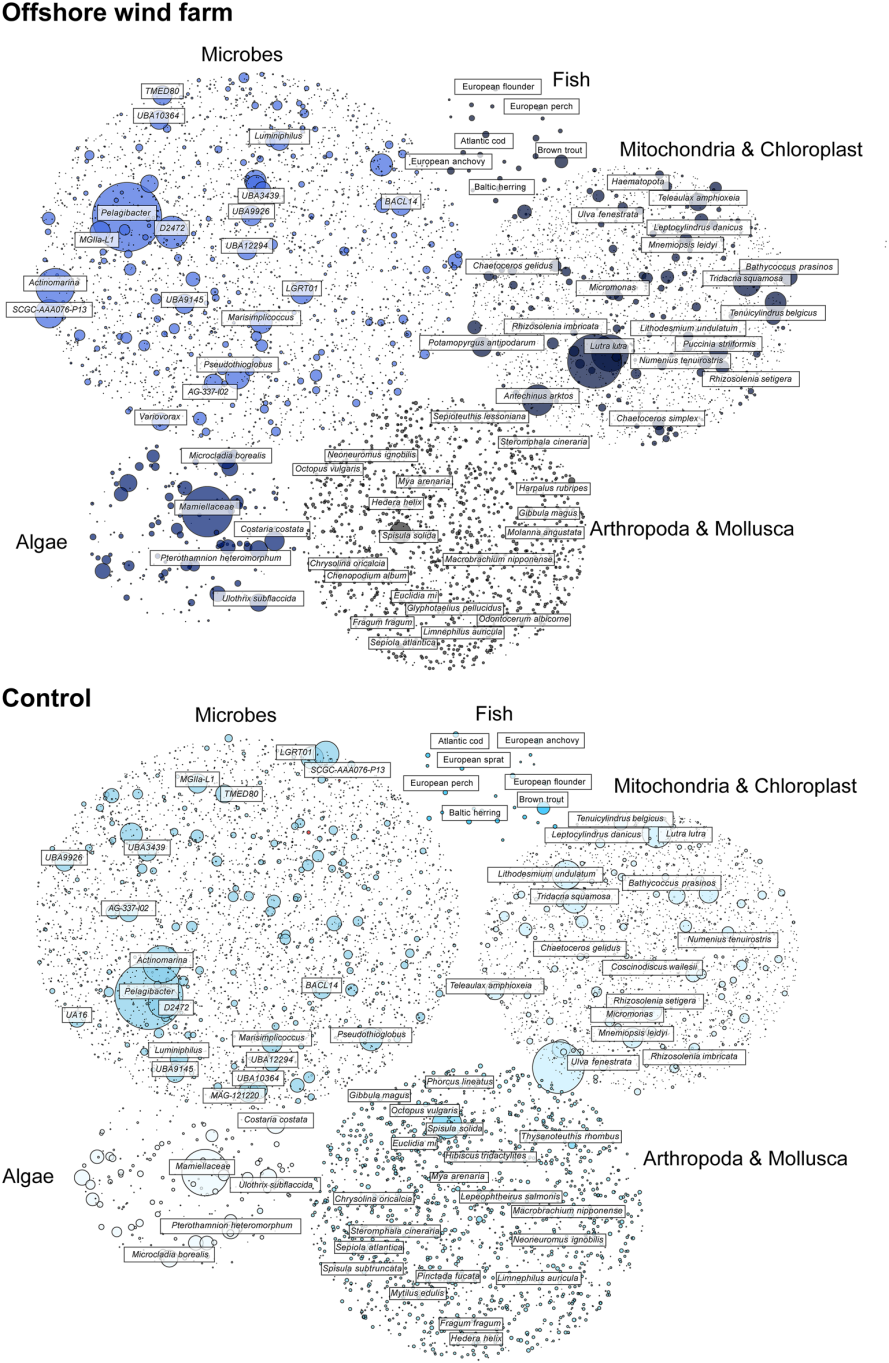
Taxonomic overview of detected species. Bubble plot illustrating the species detected in the wind farm (top) and control samples (bottom). Bubble size corresponds to the number of kmers assigned to each of these species. Species are grouped by the indicated database used for the species identification.

In total, we identified 4967 species in the samples collected inside the windfarm and 6250 species in those collected outside the windfarm (Fig. 2). Over 92% of all species were detected in all samples, and the 200 most abundant species, based on overall DNA presence, were common to all sites, indicating a high degree of congruence in biodiversity composition between the areas. The higher diversity observed in the western samples is possibly due to environmental variation rather than anthropogenic influence, as oceanographic factors, including hydrodynamics, may be stronger drivers of community structure than the wind farm presence. These findings demonstrate some of the potential of genome-scale eDNA analysis in assessing biodiversity in complex environments, providing a comprehensive snapshot of species presence and diversity without requiring direct observations or invasive sampling. While we detect genetic signatures from microbes to vertebrates, we also underscore the need for further investigation into the unclassified sequences, which may represent understudied or novel species that have an important contribution to the overall biodiversity of the ecosystem.

### Marine life detected with metagenomic eDNA analysis

Our metagenomic eDNA analysis revealed a diverse array of organisms inhabiting the waters of the Horns Rev 1 offshore wind farm. The samples were dominated by microbial DNA, with the most prominent genera being *Pelagibacter, Luminiphilus, Marine Group II, Pseudohongiella*, and *UBA10364 (Flavobacteriales)* (Fig. 2, Supplementary Tables S1 and S2). These microbial groups are of ecological importance, playing key roles in global biogeochemical cycles. For example, *Pelagibacter* species, found across both saltwater and freshwater environments, are considered major contributors to the world’s carbon cycle due to their metabolic processes^31^. *Luminiphilus*, which is widespread in saline environments, is particularly important in coastal marine ecosystems, where it occupies the euphotic zone and participates in primary production^32^. Additionally, *Marine Group II*, a group of uncultured archaea representing the most abundant planktonic archaeal group in ocean surface waters, plays a crucial role in carbon cycling in the North Sea and other marine environments^33^.

The algal communities detected, including *Botryococcus braunii*, *Microcladia borealis*, *Costaria costata*, and members of the *Mamiellaceae* family, further underscore the ecological richness of the region (Fig. 2, Supplementary Table S3). *Botryococcus braunii*, a green microalga, holds significant biotechnological interest due to its ability to produce hydrocarbons, which can be converted into biofuels^34^. The presence of *Microcladia borealis* and *Costaria costata*, both prevalent in northern latitudes, aligns with the regional environmental conditions and highlights the role of macroalgae in supporting marine biodiversity in temperate zones^35^. The detection of these previously documented algal taxa in the North Sea further demonstrates the reliability and potential of metagenomic eDNA for ecological monitoring and biodiversity assessments.

Further investigation into vertebrate biodiversity revealed the presence of several ecologically important species, including *Salmo trutta* (Brown trout), *Sprattus sprattus* (European sprat), *Clupea harengus* (Baltic herring), *Hyperoplus lanceolatus* (Great sand eel), *Gadus morhua* (Atlantic cod), and *Engraulis encrasicolus* (European anchovy) (Fig. 2, Supplementary Table S4). These species serve as vital prey for a range of marine predators and are integral to the structure of the marine food web. For example, the European sprat is a keystone species in the North Sea ecosystem, forming the basis of the diet for many larger fish, seabirds, and marine mammals^36^. The Baltic herring, another important forage fish, is a crucial prey species for predators such as cod and marine mammals, influencing predator–prey dynamics in the region^37^. The detection of Great sand eel, which inhabits sandy substrates, highlights the importance of benthic ecosystems and the sensitivity of sand eel populations to environmental changes, particularly those driven by anthropogenic activities like offshore wind farms^38^. Interestingly, the relatively high abundance detection of *Engraulis encrasicolus* in the North Sea, a species more common in warmer waters, may be an indication of its shifting distributions, linked to rising sea temperatures^39^. Such shifts in species distribution can have implications for predator–prey relationships and the overall marine ecosystem, particularly if species like the anchovy become more abundant in the North Sea as warming continues^40^.

Our analysis also revealed a high abundance of several Animalia species. Notably, we consistently detected surf clam (*Spisula solida*), Atlantic bobtail squid (*Sepiola atlantica*), sand gaper (*Mya arenaria*), and blue mussel (*Mytilus edulis*) across all the sampling sites (Supplementary Table S5). These species are of particular relevance for biodiversity monitoring. Surf clams, sand gapers, and blue mussels are important components of benthic communities and serve as indicators of substrate health and potential changes in sedimentation dynamics^41,42^. Atlantic bobtail squid, on the other hand, is a key species in the pelagic food web, with its presence and abundance reflecting changes in prey availability and broader ecosystem balance^43,44^. Monitoring these species may therefore provide valuable insights into the ecological impacts of offshore infrastructures on both benthic and pelagic habitats.

In addition to the detected vertebrate species, our metagenomic analysis revealed the consistent presence of several known bioindicator species across all samples, including *Thalassiosira*, *Phaeocystis*, and *Skeletonema* (Fig. 2, Supplementary Tables S1 and S2). *Thalassiosira*, a genus of centric diatoms, are key contributors to carbon sequestration and oxygen production in marine environments^45^. *Phaeocystis* blooms can contribute significantly to carbon cycling and the marine sulfur cycle through the production of dimethyl sulfide (DMS), a volatile compound that influences cloud formation and climate regulation^46^. Conversely, extensive blooms of *Skeletonema* can lead to hypoxic events in coastal zones, disrupting local biodiversity by depleting dissolved oxygen levels and altering habitat conditions^47^. These diatoms and algae are commonly used for the monitoring of water quality and ecosystem health due to their ecological sensitivity and biogeochemical importance. These findings also highlight the utility of metagenomics as a tool for detecting ecologically informative taxa, offering a high-resolution approach to the early identification of potential environmental stressors.

Despite the overall high correlation in species abundances between samples within and outside the windfarm, we identified a few genera that exhibited differential representation between sample sites (Supplementary Fig. S3). These included DNA classified as *Parasagitta*, a genus of arrow worms commonly found in high-latitude marine and coastal environments, and *Actinoptychus*, a genus of diatoms, which were both more abundant in samples collected outside the wind farm. In contrast, the wind farm samples showed higher relative abundances of *Fibrocapsa* (a genus of algae), *Vibrio* (Gram-negative marine bacteria, some of which are associated with foodborne and soft-tissue infections), and the fish genera *Ammodytes* and *Hyperoplus*, both characteristic of the northeastern Atlantic Ocean.

### Population genomic insights from eDNA

Our metagenomic approach enabled the extraction of population genomic insights for several detected species. While genomic DNA from multiple fish, seabird, and marine mammal species was identified (Fig. 2), taxonomic false assignments can occasionally occur due to random sequence similarity between unrelated taxa^48^. Although natural genetic variation and sequencing errors introduce some base-level divergence, misassigned reads typically show significantly greater divergence from reference genomes than true endogenous sequences. To address this, we implemented an additional filtering step based on sequence divergence. For each taxonomically assigned species, we calculated the proportion of mismatches relative to the reference genome (Supplementary Fig. S4). Species with a high divergence against the nuclear, mitochondrial or chloroplast genomes were considered unreliable and excluded from further analyses.

**Fig. 3:**
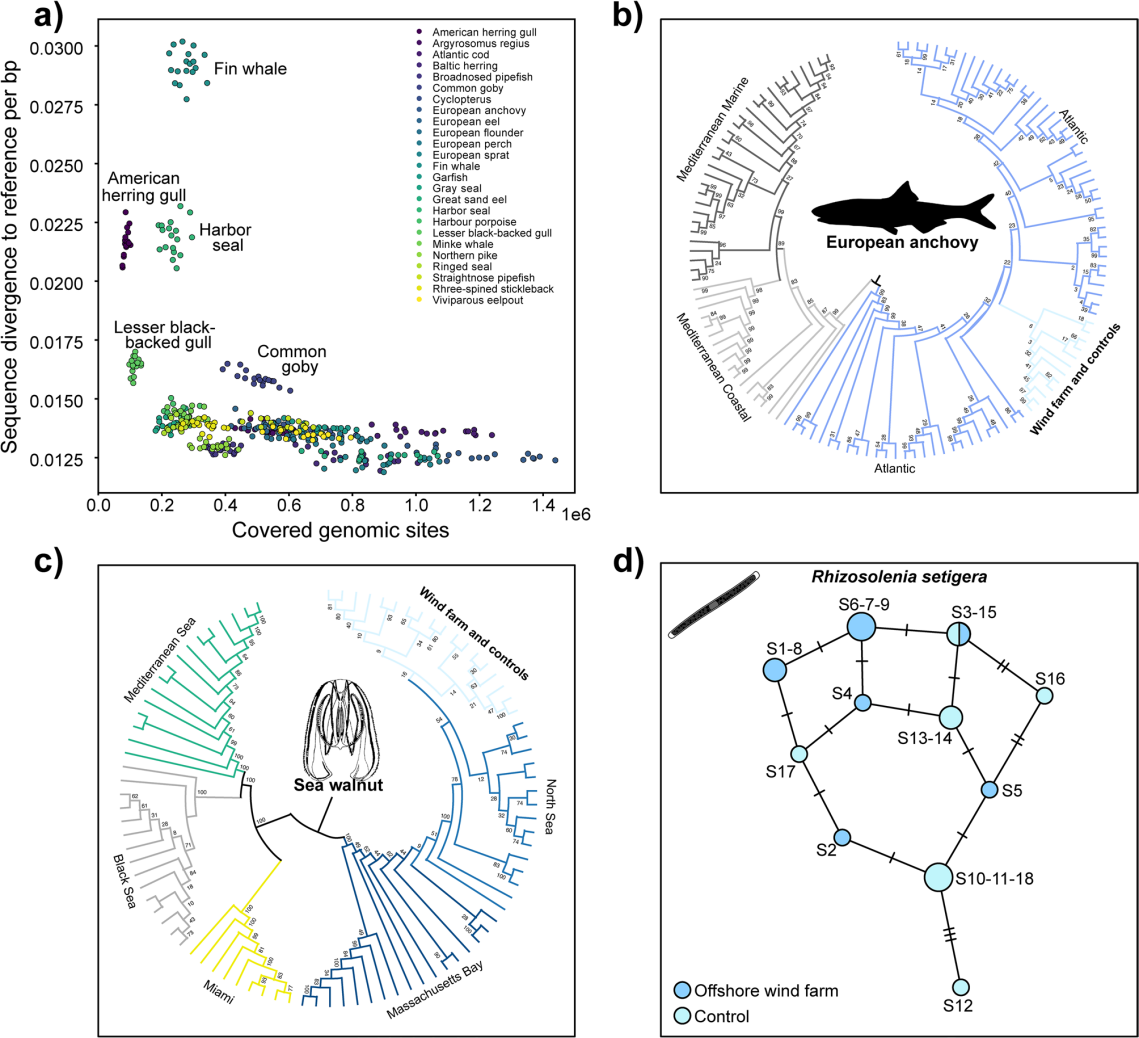
Environmental DNA-based population genomic inferences. (**a**) Number of genomic sites covered and sequence divergence between the DNA obtained from the seawater samples and their respective fish and mammalian reference genomes in the database. (**b**) Phylogenomic tree of the European anchovy and (**c**) sea walnut. The eDNA reads from water samples were aligned with previously sequenced genomes. A neighbor-joining tree was constructed based on pairwise comparisons between all detected genomic variants. (**d**) Mitochondrial haplotype network for Rhizosolenia setigera. Circles size represents the number of identified haplotypes, with crossing-lines indicating the number of base pair differences. Colors and numbers correspond to the sampling locations.

Among the taxa initially detected, species such as fin whale, harbor seal, herring gull, and common goby exhibited high sequence divergence from their respective reference genomes, indicating that these assignments were likely spurious (Fig. 3a). In contrast, several species, including European anchovy, Atlantic cod, European sprat, Great sand eel, and Baltic herring, were represented by over one million genomic bases across multiple seawater samples and showed low sequence divergence, supporting their presence within the wind farm environment with high confidence.

European anchovy (*Engraulis encrasicolus*) emerged as one of the most abundant fish species detected, with each sample containing between 1 and 1.5 million sequenced genomic bases. This high sequence coverage enabled a comparative analysis of genetic relationships between the detected DNA and reference genomes from previously sequenced Atlantic and Mediterranean populations^49^. Phylogenomic analysis revealed that the anchovy sequences from our samples clustered most closely with Atlantic populations, with some genetic affinity to the Mediterranean populations revealed by a Principal Component analysis, suggesting potential gene flow between these regions (Fig. 3b and Supplementary Fig. S5a).

Another species of particular interest was the sea walnut *Mnemiopsis leidyi*, a comb jelly native to the western Atlantic that has become invasive in European and western Asian waters^50^. *M. leidyi* is a highly opportunistic species with rapid reproduction and broad environmental tolerance, making it a significant ecological concern in non-native regions^51^. As a predator of zooplankton, fish eggs, and larvae, its introduction can disrupt local food webs, outcompete native gelatinous plankton, and contribute to declines in commercially important fish stocks^52^. By comparing the eDNA sequences from our water samples to high-coverage genomes of *M. leidyi* from across its known range^53^, we traced the origin of the North Sea population (Fig. 3c, and Supplementary Fig S5b). Our results indicated that the detected DNA closely matched *M. leidyi* genomes previously identified in the North Sea, which in turn were genetically similar to Atlantic populations from the Massachusetts coast. In contrast, individuals from Miami and the Mediterranean were genetically distinct, suggesting that the sea walnut population in the offshore wind farm originated from the northern Atlantic region. This study could serve as a basis for further research into tracking invasive species through eDNA genomics, particularly focusing on their introduction routes and their seasonal behaviors.

Finally, we obtained near-complete mitochondrial genomes for one of the most abundant diatom species in our samples, *Rhizosolenia setigera*. This allowed us to construct haplotype networks and assess population structure between the different sites (Fig. 3D). A total of 11 distinct haplotypes were identified but the haplotype network did not reveal any apparent geographic differentiation, indicating that the *R. setigera* population is genetically homogenous inside and around the wind farm. While our population genetic results indicate a high degree of connectivity between the wind farm and the surrounding marine environment, ocean currents likely play a major role in the dispersal and mixing of eDNA. The potential influence of water flow on our findings was not explicitly tested in this study, and future research could integrate hydrodynamic modeling to better distinguish localized eDNA signals from those transported by regional currents.

### Potential and limitations of eDNA metagenomics for biodiversity assessment

In this study, we employed a comprehensive metagenomic eDNA approach, combined with large reference genome databases, to investigate the marine biodiversity around the Horns Rev 1 offshore wind farm. Unlike metabarcoding, this approach does not rely on predefined genetic loci, and instead sequences all genetic material present in a sample. This allows us to capture high-resolution insights into taxonomic diversity across multiple kingdoms of life, as well as explore population-level genomic variation for several key species. Our results demonstrated the capacity of metagenomics to recover genomic information from a broad range of marine taxa, including fish, algae, molluscs, arthropods and microbial communities, and enabled investigation of population structure and gene flow, exemplified by our analysis of the European anchovy and the diatom *Rhizosolenia setigera*. In both cases, the genomic evidence suggested that the wind farm may not act as a significant barrier to genetic connectivity, aligning with prior studies showing that offshore wind farms do not obstruct fish migration and may support marine life by acting as artificial reefs^54,55^.

Additionally, we demonstrated that metagenomics can support early detection and source-tracking of invasive species, such as the sea walnut (*Mnemiopsis leidyi*), and can thus be used for ecosystem surveillance, complementing conventional monitoring strategies. However, metagenomic eDNA analysis also presents notable challenges. Despite leveraging a large reference genome database, a significant fraction of sequencing reads remained unclassified, reflecting the presence of either poorly characterized or yet-undescribed species. Furthermore, spurious read assignments, especially in cases involving low-abundance taxa, highlight the importance of stringent quality control and targeted bioinformatic filtering. Continued expansion of reference genomes and advances in classification algorithms will be essential for taxonomic resolution, reducing false positives, and minimizing the need for manual curation, ultimately streamlining the analytical workflow.

Environmental variables, such as ocean currents, also complicate interpretation. These hydrodynamic processes can transport eDNA across spatial scales, affecting the apparent distribution of species and the interpretation of population structure. While this limitation is not unique to metagenomics and applies to all eDNA-based approaches, it remains a critical consideration when using genetic material as a proxy for biodiversity assessments.

Despite these limitations, our study highlights the utility of metagenomics as a non-invasive, genome-wide approach for ecosystem monitoring. We observed a high degree of species overlap between the wind farm and surrounding sites, suggesting no major impact of the installation on overall biodiversity. The detection of bioindicator plankton species such as *Thalassiosira*, *Phaeocystis*, and *Skeletonema* further highlights the potential of metagenomics to assess water quality and marine food chain integrity.

As reference databases grow and methodologies improve, metagenomics will increasingly complement traditional monitoring methods. Future studies that incorporate hydrodynamic modeling and seasonal sampling will help refine our understanding of eDNA transport and persistence, ultimately enhancing the spatial and temporal resolution of biodiversity assessments in offshore and other dynamic marine environments.

## Material and methods

### Field sampling

Sampling was conducted in October 2023 at the Horns Rev 1 Offshore Wind Farm, located on the western coast of Jutland, Denmark. A total of 34 seawater samples were collected at approximately 10 cm depth below the surface, directly adjacent to wind turbines across the wind farm. An additional 28 samples were collected from control sites positioned at approximately 500-m intervals to the east, west, and south of the wind farm, spanning distances of 500 to 5000 m from its perimeter in each direction (Supplementary Table S11). Due to shallow water depths and limited accessibility, no samples were obtained north of the wind farm. From each sampling location, a single 100 mL seawater sample was collected by manually submerging a sterile container just below the surface and scooping the water. Sampling was conducted from October 26 to 29, 2023, using maintenance and crew transfer vessels, during which sea surface temperatures ranged from 16.9 to 17.1 °C. All samples were immediately frozen at –20 °C for subsequent processing.

### DNA extraction, library preparation, and shotgun sequencing

Samples were pooled based on their geographical proximity prior to DNA extraction. For each pool, equal volumes of seawater from individual samples were combined to obtain a total pooled volume of 50 mL, corresponding to the number of individual samples included in each pool. Each pooled sample was then concentrated from 50 mL to 200 µL using ultrafiltration (3 kDa Amicon® Ultra Centrifugal Filter), then subjected to total DNA extraction using the Qiagen DNeasy Blood and Tissue Kit (Qiagen) following the manufacturer’s protocol. Extracted DNA was fragmented to an average size of 350 bp via sonication (Covaris). For each pool, double-indexed DNA libraries were prepared using a previous protocol^56^, and sequenced on a single lane of the NovaSeq X platform at SciLifeLab (Sweden). An additional extraction blank sample were sequenced to quantify possible laboratory contamination.

### Bioinformatic and statistical analyses

Each sample pool was sequenced to between 59 and 97 million reads. After removing sequencing adapters and low-quality reads, all pools were randomly downsampled to 50 million reads for downstream analysis using *seqkit sample -n 50.000.000*^57^. This step was implemented to mitigate potential biases from variations in sequencing depth and to ensure comparability across the sample pools. The blank sample yielded less than 20,000 reads, demonstrating that the contamination rates among the samples was likely below 0.04%.

All sequence data was adapter trimmed and subsequently overlapping read pairs were merged using *fastp v0.23.4*^58^. *Jellyfish v2.3.0* was then used to calculate the total number of unique 21 base pair sequences (21-mers) in each of the sequence sample pools using the command *jellyfish count -C -m 21 sample.fastq.gz*. The total number of unique 21-mers was then divided by the total number of reads (50 million), providing a global estimate of the number of unique sequences in each of the samples. Next, Mash was used to obtain genetic distance estimates between all samples^59^. Clustering of the samples was then conducted using Python v3. Scipy.cluster.hierarchy^60^.

To classify sequencing reads taxonomically, a custom Kraken2 v2.1.3 database^61^ was first constructed to incorporate all available genomes from the Genome Taxonomy Database^62^. This microbial database includes complete genomes for 584,382 bacterial species and 12,477 archaeal species. Kraken2 was then run with default parameters to classify all sequencing reads against this database. Reads that remained unclassified were subsequently analyzed using the complete NCBI RefSeq Kraken2 database (version 2024-May), which includes reference sequences for both microbial and eukaryotic taxa. In addition, a third custom reference index was constructed for Bowtie2 v2.5.2^63^, comprising all RefSeq mitochondrial genomes (17,634 species), all RefSeq chloroplast genomes (14,515 species), complete genomes of algae (270 species) and complete genomes of fish and aquatic mammals (25 species) that are known to occur in the Baltic Sea (Supplementary Tables S1-S10). All these genomes were concatenated into a single reference file and indexed using *Bowtie2 v2.5.2*^63^. Reads not classified as bacterial were aligned to this index using end-to-end Bowtie2 alignments with the *–very-sensitive* parameter. All reads that could be aligned to more than one species were all removed.

To compare the abundance of classified DNA across the different databases (microbial, eukaryotic, mitochondrial/chloroplast, and aquatic animal genomes), the number of assigned k-mers was quantified for the Kraken2-classified microbial and eukaryotic datasets, and the number of uniquely aligned reads for the mitochondrial/chloroplast and aquatic animal genome datasets. Correlation coefficients between the assigned data to each species in the control and windfarm samples were then calculated using the Pearson product-moment correlation coefficients in Python v3.

The mismatch rate per base pair between the mapped reads and the reference genomes was calculated using ANGSD dofasta v0.940 with parameters *-doFasta 1 -doCounts 1 -remove_bads 1 -minMapQ 20 -uniqueOnly 1*, sampling a random base at each site covered by at least one read^64^. To perform principal component analysis (PCA), high-quality genomes of the species of interest were downloaded from ENA and mapped to the Bowtie2 index using end-to-end Bowtie2 alignments with the *–very-sensitive* parameter. A random allele was called for each mapped genomic site using ANGSD haplocaller v0.940 with parameters: *-doCounts 1 -dohaplocall 1 -minMinor 1 -remove_bads 1 -minMapQ 20 -uniqueOnly 1*^64^. The resulting output files were converted to VCF and PLINK BED format^65^. PCA was then performed using PCAngsd v1.2, optimized for low-coverage samples, to assess genetic relationships between the samples and previously sequenced genomes^66^. Phylogenomic trees were constructed using the output files from ANGSD dofasta and subsequently obtaining the optimal phylogenomic model and maximum likelihood tree with IQtree v2.4.0 on default parameters^67^.

For the diatom species *Rhizosolenia setigera*, mitochondrial genomes were reconstructed by calling the majority allele at each mitochondrial site using ANGSD dofasta with the following parameters: *-doFasta 1 -doCounts 1 -remove_bads 1 -uniqueOnly 1 -minMapQ 20*. A haplotype network of the resulting FASTA alignment was then constructed using PopArt^68^.

## Supplementary Information

Supplementary Information 1.

Supplementary Information 2.

## Data Availability

All sequence data generated in this study have been deposited in the European Nucleotide Archive with the primary accession code PRJEB80220.

## References

[1] International Energy Agency. *World Energy Outlook 2019*. (2019).

[2] Dean, N. Performance factors. *Nat. Energy* **5**, 5–5 (2020).

[3] Bergström, L. et al. Effects of offshore wind farms on marine wildlife—a generalized impact assessment. *Environ. Res. Lett.* **9**, 034012 (2014).

[4] Bailey, H., Brookes, K. L. & Thompson, P. M. Assessing environmental impacts of offshore wind farms: lessons learned and recommendations for the future. *Aquat. Biosyst.* **10**, 8 (2014). 25250175 10.1186/2046-9063-10-8 PMC4172316

[5] Galparsoro, I. et al. Reviewing the ecological impacts of offshore wind farms. *npj Ocean Sustain.* **1**, 1–8 (2022).

[6] Hall, R., João, E. & Knapp, C. W. Environmental impacts of decommissioning: Onshore versus offshore wind farms. *Environ. Impact Assess. Rev.* **83**, 106404 (2020).

[7] Vaissière, A.-C., Levrel, H., Pioch, S. & Carlier, A. Biodiversity offsets for offshore wind farm projects: The current situation in Europe. *Mar. Policy* **48**, 172–183 (2014).

[8] Wilhelmsson, D., Malm, T. & Öhman, M. C. The influence of offshore windpower on demersal fish. *ICES J. Mar. Sci.* **63**, 775–784 (2006).

[9] Scheidat, M. et al. Harbour porpoises (Phocoena phocoena) and wind farms: a case study in the Dutch NorthSea. *Environ. Res. Lett.* **6**, 025102 (2011).

[10] Glarou, M., Zrust, M. & Svendsen, J. C. Using artificial-reef knowledge to enhance the ecological function of offshore wind turbine foundations: Implications for fish abundance and diversity. *J. Mar. Sci. Eng.* **8**, 332 (2020).

[11] ter Hofstede, R., Driessen, F. M. F., Elzinga, P. J., Van Koningsveld, M. & Schutter, M. Offshore wind farms contribute to epibenthic biodiversity in the North Sea. *J. Sea Res.* **185**, 102229 (2022).

[12] Gimpel, A. et al. Ecological effects of offshore wind farms on Atlantic cod (Gadus morhua) in the southern North Sea. *Sci. Total Environ.* **878**, 162902 (2023). 36934919 10.1016/j.scitotenv.2023.162902

[13] Lindeboom, H. J. et al. Short-term ecological effects of an offshore wind farm in the Dutch coastal zone; a compilation. *Environ. Res. Lett.* **6**, 035101 (2011).

[14] Svendsen, J. C. et al. Effects of operational off-shore wind farms on fishes and fisheries. Review report. DTU Aqua. DTU Aqua-rapport No. 411-2022 (2022).

[15] Garthe, S., Markones, N. & Corman, A.-M. Possible impacts of offshore wind farms on seabirds: a pilot study in Northern Gannets in the southern North Sea. *J. Ornithol.* **158**, 345–349 (2017).

[16] Thomsen, P. F. & Willerslev, E. Environmental DNA – An emerging tool in conservation for monitoring past and present biodiversity. *Biol. Conserv.* **183**, 4–18 (2015).

[17] Taberlet, P., Coissac, E., Hajibabaei, M. & Rieseberg, L. H. Environmental DNA. *Mol. Ecol.* **21**, 1789–1793 (2012). 22486819 10.1111/j.1365-294X.2012.05542.x

[18] Yang, H. et al. Effectiveness assessment of using riverine water eDNA to simultaneously monitor the riverine and riparian biodiversity information. *Sci. Rep.* **11**, 24241 (2021). 34930992 10.1038/s41598-021-03733-7 PMC8688430

[19] Jerney, J. et al. *DNA Metabarcoding: Guidelines to Monitor Phytoplankton Diversity and Distribution in Marine and Brackish Waters* (Nordic Council of Ministers, 2023).

[20] Wilms, T. J. G. et al. Environmental DNA reveals fine-scale habitat associations for sedentary and resident marine species across a coastal mosaic of soft- and hard-bottom habitats. *Environ. DNA* **4**, 954–971 (2022).

[21] Boyse, E. et al. Environmental DNA reveals fine-scale spatial and temporal variation of marine mammals and their prey species in a Scottish marine protected area. *Environ. DNA*10.1002/edn3.587 (2024).

[22] Shokralla, S., Spall, J. L., Gibson, J. F. & Hajibabaei, M. Next-generation sequencing technologies for environmental DNA research. *Mol. Ecol.* **21**, 1794–1805 (2012). 22486820 10.1111/j.1365-294X.2012.05538.x

[23] Adams, C. I. M. et al. Beyond biodiversity: Can environmental DNA (eDNA) cut it as a population genetics tool?. *Genes* **10**, 192 (2019). 30832286 10.3390/genes10030192 PMC6470983

[24] Cutler, D. J. & Jensen, J. D. To pool, or not to pool?. *Genetics* **186**, 41–43 (2010). 20855575 10.1534/genetics.110.121012 PMC2940305

[25] Zepeda Mendoza, M. L., Sicheritz-Pontén, T. & Gilbert, M. T. P. Environmental genes and genomes: understanding the differences and challenges in the approaches and software for their analyses. *Brief Bioinform.* **16**, 745–758 (2015). 25673291 10.1093/bib/bbv001 PMC4570204

[26] Semenov, M. V. Metabarcoding and metagenomics in soil ecology research: Achievements, challenges, and prospects. *Biol. Bull. Rev.* **11**, 40–53 (2021).

[27] Beng, K. C. & Corlett, R. T. Applications of environmental DNA (eDNA) in ecology and conservation: opportunities, challenges and prospects. *Biodivers. Conserv.* **29**, 2089–2121 (2020).

[28] Taberlet, P., Bonin, A., Zinger, L. & Coissac, E. *Environmental DNA: For Biodiversity Research and Monitoring* (Oxford University Press, 2018).

[29] Rieder, J., Kapopoulou, A., Bank, C. & Adrian-Kalchhauser, I. Metagenomics and metabarcoding experimental choices and their impact on microbial community characterization in freshwater recirculating aquaculture systems. *Environ. Microbiome* **18**, 8 (2023). 36788626 10.1186/s40793-023-00459-z PMC9930364

[30] Kihlström L-M. How Horns Rev 1 paved the way for offshore wind. *Vattenfall* https://group.vattenfall.com/press-and-media/newsroom/2023/how-horns-rev-1-paved-the-way-for-offshore-wind. (2023).

[31] Rappé, M. S., Connon, S. A., Vergin, K. L. & Giovannoni, S. J. Cultivation of the ubiquitous SAR11 marine bacterioplankton clade. *Nature* **418**, 630–633 (2002). 12167859 10.1038/nature00917

[32] Spring, S. et al. Taxonomy and evolution of bacteriochlorophyll a-containing members of the OM60/NOR5 clade of marine gammaproteobacteria: description of Luminiphilus syltensis gen. nov., sp. nov., reclassification of Haliea rubra as Pseudohaliea rubra gen. nov., comb. nov., and emendation of Chromatocurvus halotolerans. *BMC Microbiol.* **13**, 118 (2013). 23705883 10.1186/1471-2180-13-118 PMC3679898

[33] Zhang, C. L., Xie, W., Martin-Cuadrado, A.-B. & Rodriguez-Valera, F. Marine group II archaea, potentially important players in the global ocean carbon cycle. *Front. Microbiol.* **6**, 1108 (2015). 26528260 10.3389/fmicb.2015.01108 PMC4602124

[34] Metzger, P. & Largeau, C. Botryococcus braunii: a rich source for hydrocarbons and related ether lipids. *Appl. Microbiol. Biotechnol.* **66**, 486–496 (2005). 15630516 10.1007/s00253-004-1779-z

[35] Wiencke, C. & Bischof, K. *Seaweed Biology: Novel Insights into Ecophysiology Ecology and Utilization* (Springer Science & Business Media, 2012).

[36] Harvey, C. J., Cox, S. P., Essington, T. E., Hansson, S. & Kitchell, J. F. An ecosystem model of food web and fisheries interactions in the Baltic Sea. *ICES J. Mar. Sci.* **60**, 939–950 (2003).

[37] Andersson, L., André, C., Johannesson, K. & Pettersson, M. Ecological adaptation in cod and herring and possible consequences of future climate change in the Baltic Sea. *Front. Mar. Sci.*10.3389/fmars.2023.1101855 (2023).

[38] Sherman, K. et al. Congruent shifts in sand eel abundance in western and eastern North Atlantic ecosystems. *Nature* **291**, 486–489 (1981).

[39] Petitgas, P. et al. Anchovy population expansion in the North Sea. *Mar. Ecol. Prog. Ser.* **444**, 1–13 (2012).

[40] Raybaud, V., Bacha, M., Amara, R. & Beaugrand, G. Forecasting climate-driven changes in the geographical range of the European anchovy (Engraulis encrasicolus). *ICES J. Mar. Sci.* **74**, 1288–1299 (2017).

[41] Dolbeth, M., Viegas, I., Martinho, F., Marques, J. C. & Pardal, M. A. Population structure and species dynamics of Spisula solida, Diogenes pugilator and Branchiostoma lanceolatum along a temporal–spatial gradient in the south coast of Portugal. *Estuar. Coast. Shelf Sci.* **66**, 168–176 (2006).

[42] Beyer, J. et al. Blue mussels (Mytilus edulis spp.) as sentinel organisms in coastal pollution monitoring: A review. *Mar. Environ. Res.* **130**, 338–365 (2017). 28802590 10.1016/j.marenvres.2017.07.024

[43] Yau, C. & Boyle, P. R. Ecology of Sepiola atlantica (Mollusca: Cephalopoda) in the shallow sublittoral zone. *J. Mar. Biol. Assoc. U. K.* **76**, 733–748 (1996).

[44] Drerup, C. The behavioural ecology of Sepiolidae (Cephalopoda: Sepiolida): a review. *Molluscan Res.* **42**, 185–204 (2022).

[45] Chappell, P. D. et al. Thalassiosira spp. community composition shifts in response to chemical and physical forcing in the northeast Pacific Ocean. *Front. Microbiol.* **4**, 273 (2013). 24065961 10.3389/fmicb.2013.00273 PMC3779818

[46] Stefels, J., Dijkhuizen, L. & Gieskes, W. W. C. DMSP-lyase activity in a spring phytoplankton bloom off the Dutch coast, related to Phaeocystis sp. abundance. *Mar. Ecol. Prog. Ser.* **123**, 235–243 (1995).

[47] Peperzak, L. Climate change and harmful algal blooms in the North Sea. *Acta Oecol.* **24**, S139–S144 (2003). 15918356

[48] Oskolkov, N. et al. Disinfecting eukaryotic reference genomes to improve taxonomic inference from ancient environmental metagenomic data. *bioRxiv*10.1101/2025.03.19.644176 (2025).

[49] Bonhomme, F. et al. Systematics of European coastal anchovies (genus Engraulis Cuvier). *J. Fish Biol.* **100**, 594–600 (2022). 34837218 10.1111/jfb.14964

[50] Javidpour, J., Sommer, U. & Shiganova, T. First record of Mnemiopsis leidyi A. Agassiz 1865 in the Baltic Sea. *Aquat. Invasions* **1**, 299–302 (2006).

[51] Costello, J. H., Bayha, K. M., Mianzan, H. W., Shiganova, T. A. & Purcell, J. E. Transitions of Mnemiopsis leidyi (Ctenophora: Lobata) from a native to an exotic species: a review. *Hydrobiologia* **690**, 21–46 (2012).

[52] Purcell, J. E., Uye, S. & Lo, W. Anthropogenic causes of jellyfish blooms and their direct consequences for humans: a review. *Mar. Ecol. Prog. Ser.* **350**, 153–174 (2007).

[53] Jaspers, C. et al. Invasion genomics uncover contrasting scenarios of genetic diversity in a widespread marine invader. *Proc. Natl. Acad. Sci. U. S. A.* **118**, e2116211118 (2021). 34911766 10.1073/pnas.2116211118 PMC8713979

[54] Stenberg, C. et al. Long-term effects of an offshore wind farm in the North Sea on fish communities. *Mar. Ecol. Prog. Ser.* **528**, 257–265 (2015).

[55] Bergström, L., Sundqvist, F. & Bergström, U. Effects of an offshore wind farm on temporal and spatial patterns in the demersal fish community. *Mar. Ecol. Prog. Ser.* **485**, 199–210 (2013).

[56] Meyer, M. & Kircher, M. Illumina sequencing library preparation for highly multiplexed target capture and sequencing. *Cold Spring Harb. Protoc.*10.1101/pdb.prot5448 (2010). 20516186 10.1101/pdb.prot5448

[57] Shen, W., Le, S., Li, Y. & Hu, F. SeqKit: A cross-platform and ultrafast toolkit for FASTA/Q file manipulation. *PLoS ONE* **11**, e0163962 (2016). 27706213 10.1371/journal.pone.0163962 PMC5051824

[58] Chen, S., Zhou, Y., Chen, Y. & Gu, J. fastp: an ultra-fast all-in-one FASTQ preprocessor. *Bioinformatics* **34**, i884–i890 (2018). 30423086 10.1093/bioinformatics/bty560 PMC6129281

[59] Ondov, B. D. et al. Mash: fast genome and metagenome distance estimation using MinHash. *Genome Biol.*10.1186/s13059-016-0997-x (2016). 27323842 10.1186/s13059-016-0997-x PMC4915045

[60] Virtanen, P. et al. SciPy 1.0: fundamental algorithms for scientific computing in Python. *Nat. Methods* **17**, 261–272 (2020). 32015543 10.1038/s41592-019-0686-2 PMC7056644

[61] Wood, D. E., Lu, J. & Langmead, B. Improved metagenomic analysis with Kraken 2. *Genome Biol.* **20**, 257 (2019). 31779668 10.1186/s13059-019-1891-0 PMC6883579

[62] Parks, D. H. et al. GTDB: an ongoing census of bacterial and archaeal diversity through a phylogenetically consistent, rank normalized and complete genome-based taxonomy. *Nucleic Acids Res.* **50**, D785–D794 (2022). 34520557 10.1093/nar/gkab776 PMC8728215

[63] Langmead, B. & Salzberg, S. L. Fast gapped-read alignment with Bowtie 2. *Nat. Methods* **9**, 357–359 (2012). 22388286 10.1038/nmeth.1923 PMC3322381

[64] Korneliussen, T. S., Albrechtsen, A. & Nielsen, R. ANGSD: Analysis of next generation sequencing data. *BMC Bioinform.* **15**, 356 (2014). 10.1186/s12859-014-0356-4 PMC4248462 25420514

[65] Purcell, S. et al. PLINK: A tool set for whole-genome association and population-based linkage analyses. *Am. J. Hum. Genet.*10.1086/519795 (2007). 17701901 10.1086/519795 PMC1950838

[66] Meisner, J., Liu, S., Huang, M. & Albrechtsen, A. Large-scale inference of population structure in presence of missingness using PCA. *Bioinformatics*10.1093/bioinformatics/btab027 (2021). 33459779 10.1093/bioinformatics/btab027

[67] Minh, B. Q. et al. IQ-TREE 2: New models and efficient methods for phylogenetic inference in the genomic era. *Mol. Biol. Evol.* **37**, 1530–1534 (2020). 32011700 10.1093/molbev/msaa015 PMC7182206

[68] Leigh, J. W. & Bryant, D. Popart: Full-feature software for haplotype network construction. *Methods Ecol. Evol.* **6**, 1110–1116 (2015).

