# Supplementary material for: Metagenomic biodiversity assessment within an offshore wind farm
Source: Sci Rep. 2025 May 14;15:16786. doi: 10.1038/s41598-025-01541-x (PMC12078662; doi:10.1038/s41598-025-01541-x)
Supplement: Supplementary file 1 — Supplementary Information 1. [file 41598_2025_1541_MOESM1_ESM.pdf]

Supplementary materials for:

Metagenomic Biodiversity Assessment of an Offshore Wind Farm

Phanu Theodore Serivichyaswat, Thijs Scholte, Tim Wilms, Liv Stranddorf, Tom van der Valk

This PDF includes:

Figure S1	2
Figure S2	3
Figure S3	4
Figure S4	5
Figure S5	6

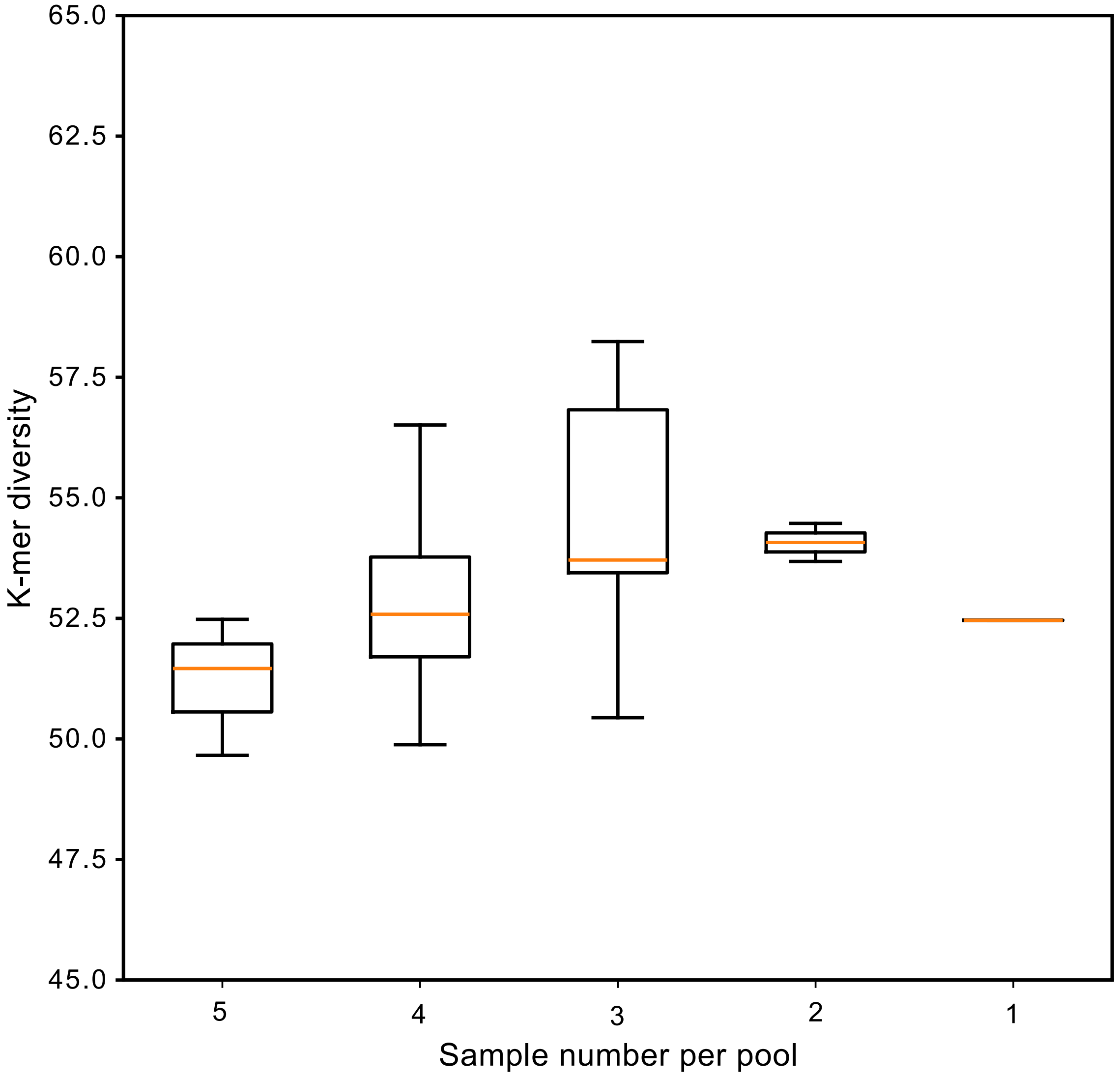

Fig. S1. *Effect of sample number per pool on diversity metrics. No significant differences were observed between pools containing different numbers of input seawater samples (values represent mean ± SD; Student's t-test).*

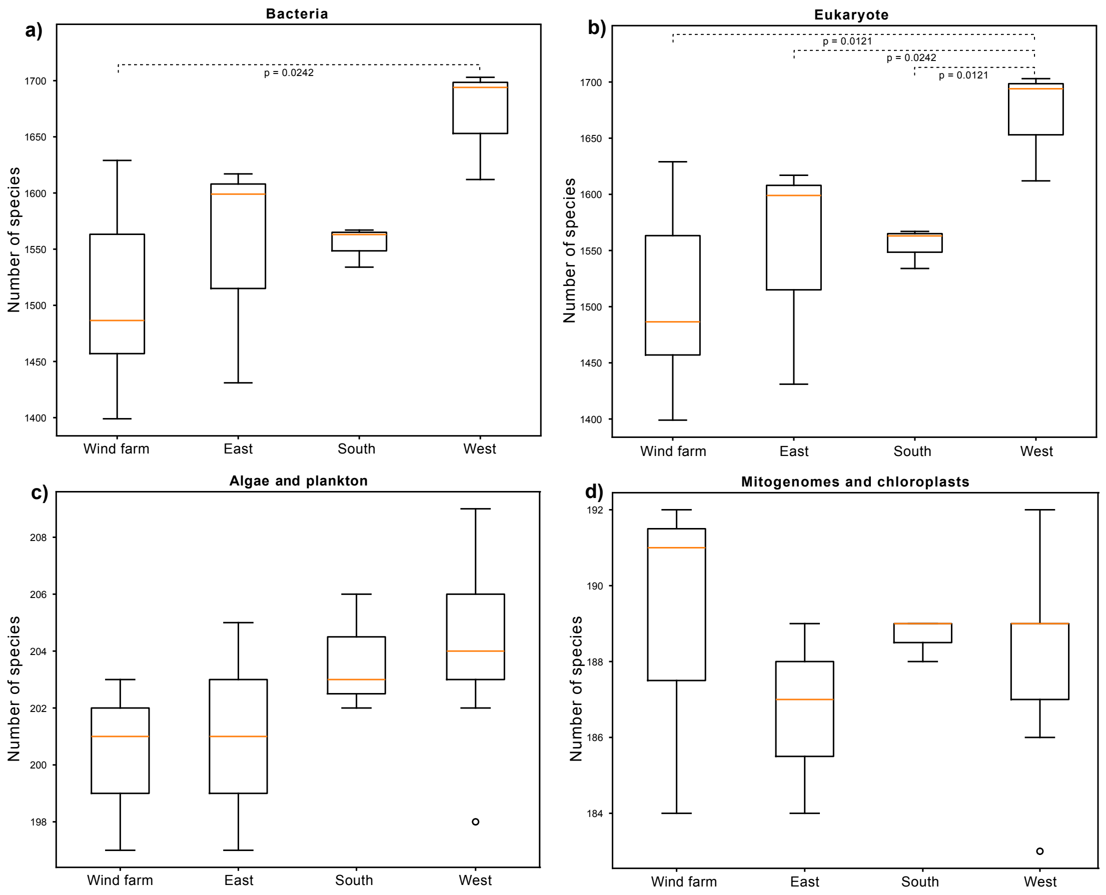

Fig. S2. *Number of species detected (after quality filtering), grouped by bacteria (a), eukaryote (b), algae and plankton (c), and mitochondria (d) datasets (values represent mean ± SD; Student's t-test).*

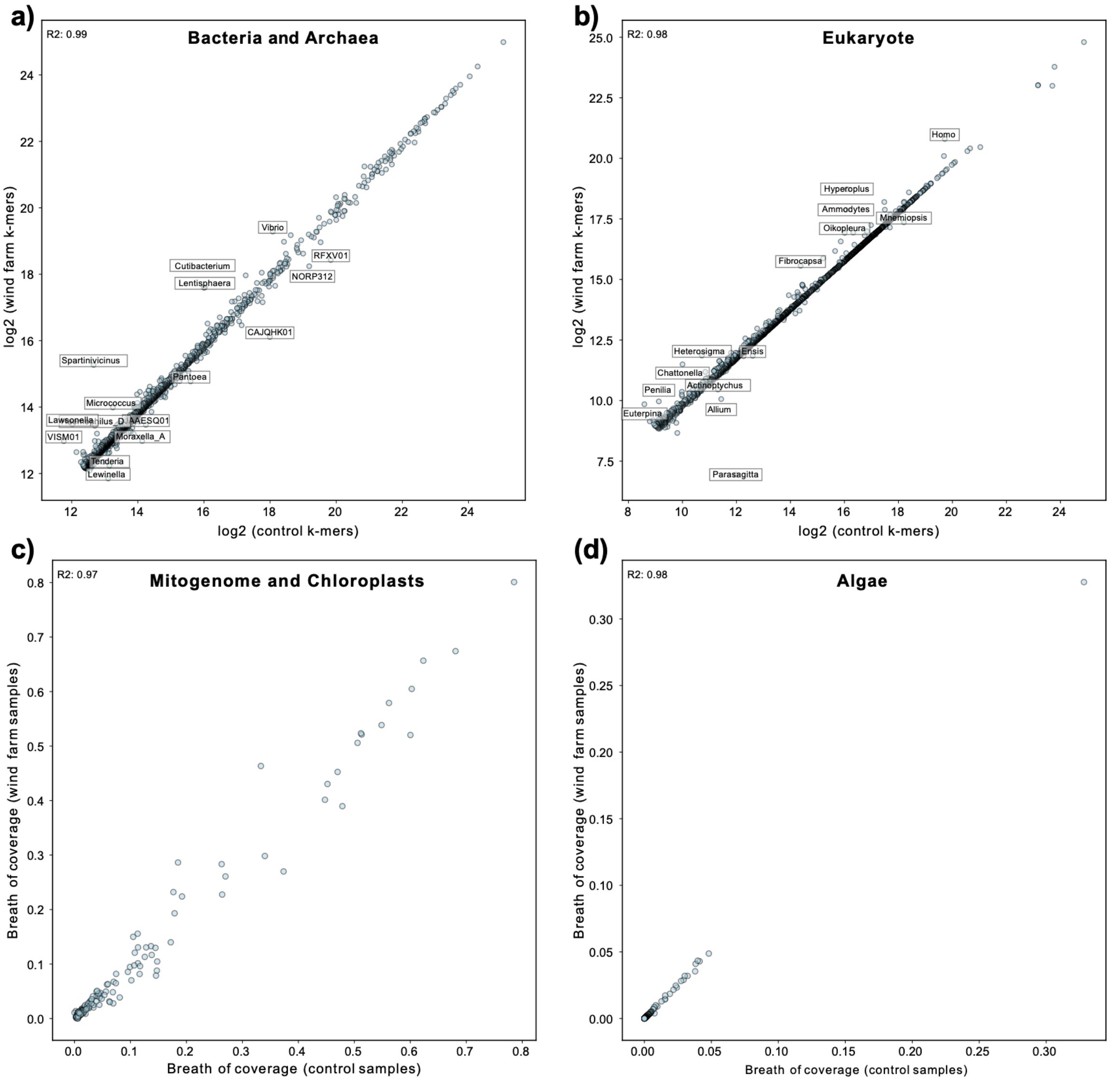

***Fig. S3*. Correlation of DNA abundance between control and wind farm samples for all detected species.** *The x-axis shows the number of identified kmers for a species, summed over all control samples and normalized for read numbers. The y-axis shows the corresponding k-mer counts in wind farm samples, also normalized. Each point represents a species, grouped into Bacteria/Archaea (a), Eukaryotes (b), mitochondrial genomes (c), and algal genomes (d). Species with significantly different abundances between control and wind farm samples are labelled.*

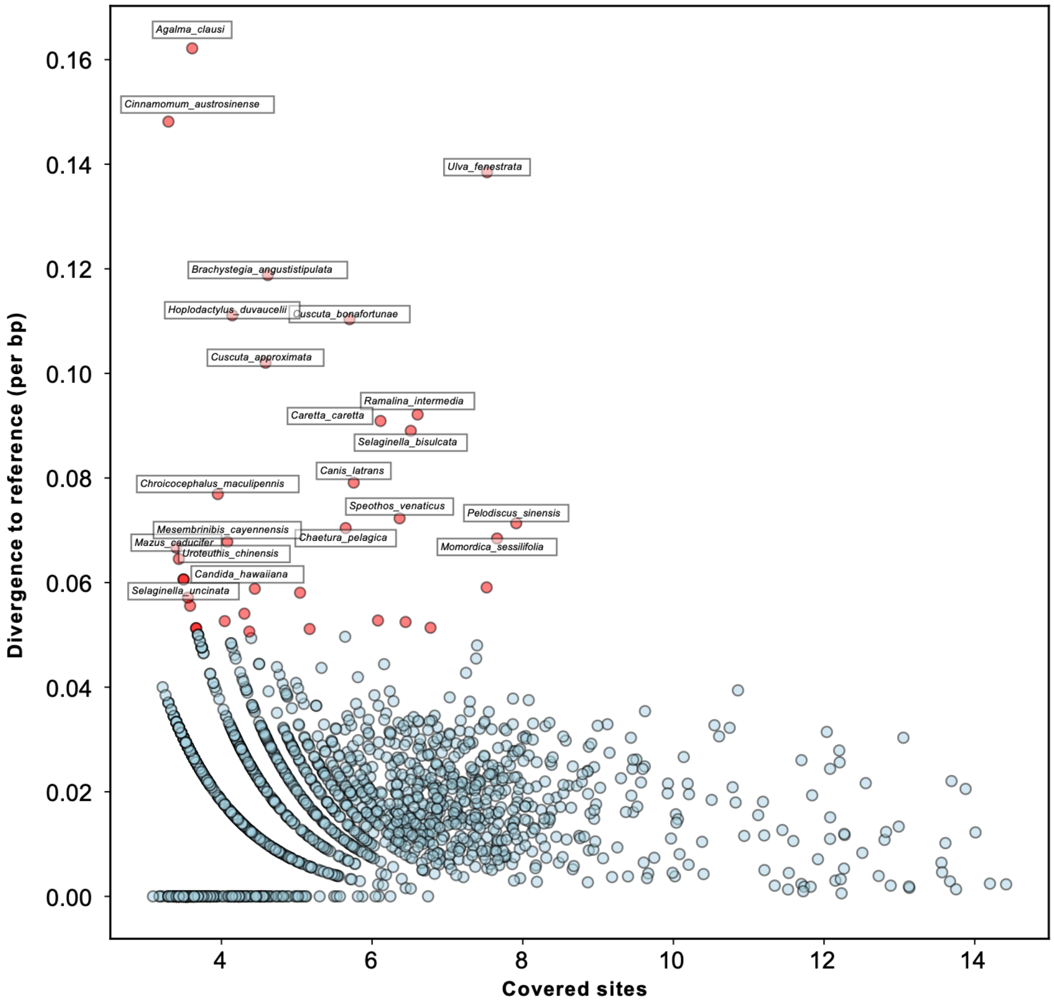

Fig. S4. *Sequence divergence of each species relative to the reference genomes, based on alignments against the mitochondrial and chloroplast reference databases. Species exhibiting greater than 5% divergence were considered likely false positives and excluded from downstream analyses.*

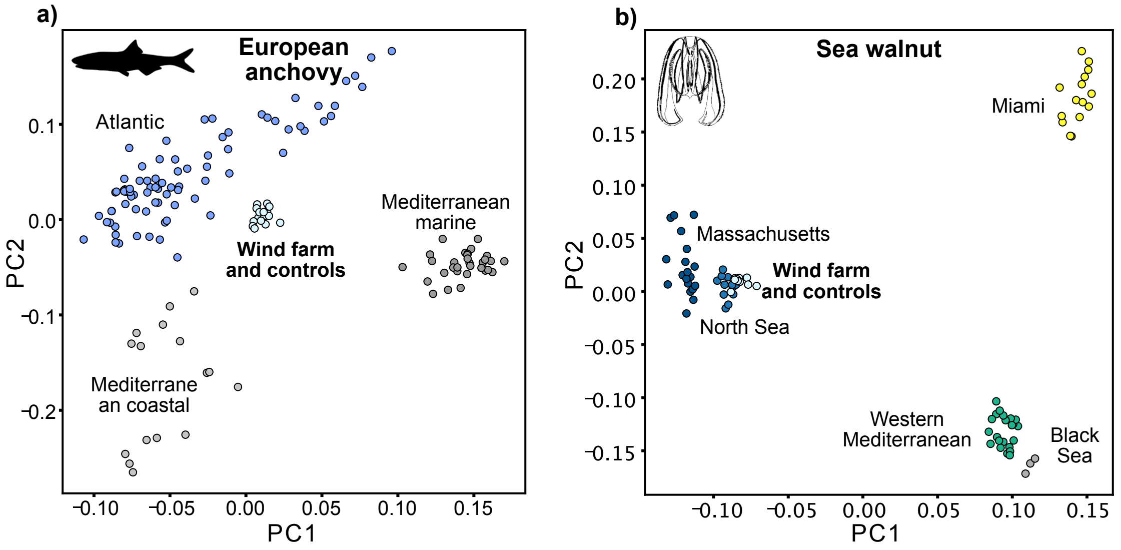

Fig. S5. *Principal component analysis (PCA) comparing eDNA sequences of (a) European anchovy and (b) sea walnut to previously sequenced genomes.*
